# A Rabies Virus Nucleocapsid-like Nanostructure Vaccine Based on Dual-Cationic Lipid Nanoparticles

**DOI:** 10.3390/vaccines13121196

**Published:** 2025-11-26

**Authors:** Zhixiao Zhang, Jingjing Zhang, Changyong Mu, Kaili Ma, Dongxiu Gao, Chang’e Liu, Lin Feng, Xiaowu Peng, Junbo Si, Hongbing Li, Yanrui Su, Fengyuan Zeng, Liping He, An Wang, Chongying Zhou, Zhenxiao Zhang, Yixuan Wang, Qiuqi Li, Jiahui Li, Shuiyan Zou, Miaomiao Xing, Huijuan Li, Meng Sun, Weijie Chang, Xiaoxia Yu, Junqing Li, Lichun Wang, Yanmei Li, Hongkun Yi, Lichun Zheng, Fuyun He, Qihan Li

**Affiliations:** 1 Weirui Biotechnology (Kunming) Co., Ltd., Ciba Biotechnology Innovation Center, Kunming 650000, China; zhixiaozhang@126.com (Z.Z.);; 2 Shandong Weigao Litong Biological Products Co., Ltd., Weihai 264200, China

**Keywords:** rabies virus, lipid nanoparticles, nucleocapsid, mRNA vaccine

## Abstract

**Background:** Rabies virus (RABV) causes approximately 59,000 human deaths annually. Current pre- and post-exposure vaccination relies on inactivated vaccines (INVs) with limited yield and immunogenicity. We engineered a dual-cationic LNP-based nucleocapsid-like nanostructure (NLS) that co-encapsulates RABV G-mRNA and recombinant RABV-N to engage MHC-I/II pathways and enhance protection. **Methods:** A pVAX-RABV-G plasmid containing 5′/3′UTRs, Kozak, and poly(A) was transcribed in vitro. RABV-N with an N-terminal 6× His tag was expressed in *E. coli* BL21(DE3) and purified by Ni-Sepharose affinity chromatography. Dual-cationic LNPs (DHA, DOTAP Cl, mPEG-DTA2K, DOPC) were formulated by microfluidics at a 4:1 (G-mRNA:RABV-N) mass ratio. Vaccine quality was assessed by encapsulation efficiency, DLS, PDI, zeta potential, and TEM. Mice received empty LNPs, INV, G-mRNA, or NLS under varied schedules and doses. ELISA measured RABV-G/N-IgG; RFFIT determined neutralizing antibody (nAb) titers; ELISPOT quantified CTL response; qPCR assessed T-cell activation genes. On day 35 after the first immunization of vaccines, mice were challenged intramuscularly with 25 LD_50_ of CVS-24. **Results:** G-mRNA purity was >95% and drove strong RABV-G expression in 293T cells. Purified RABV-N was approximately 52 kDa, >90% pure, and reactive to anti-His and anti-N antibodies. NLS achieved >95% encapsulation, a diameter of 136.9 nm, PDI 0.09, and a +18.7 mV zeta potential. A single dose yielded approximately 10 IU mL^−1^ nAb by day 7; two doses peaked at approximately 1000 IU mL^−1^. Mice showed 100% survival and no viral rebound in brain, spinal cord, and sciatic nerve. NLS induced stronger MHC-I/II-linked cellular immunity and higher RABV G/N-specific IFN-γ spot frequencies than G-mRNA or INV. **Conclusions:** The dual-antigen NLS vaccine co-delivering G-mRNA and RABV-N via dual-cationic LNPs robustly activates MHC-I/II, rapidly generates high-titer nAb (≥10 IU mL^−1^ within 1 week), and sustains potent CD8^+^ CTL and CD4^+^ Th responses. A two-dose regimen (days 0 and 21) conferred complete protection, supporting the NLS platform as a next-generation rabies vaccine candidate.

## 1. Introduction

Rabies virus (RABV) belongs to the genus *Lyssavirus*, family *Rhabdoviridae* [1,2]. It is a single-negative-stranded RNA virus with a genome of approximately 12,000 nucleotides. Its structure mainly comprises the glycoprotein (G), matrix protein (M), phosphoprotein (P) and RNA polymerase (L), together with the nucleoprotein (N) and the viral RNA encapsulated by the N protein [3,4,5,6].

RABV is a zoonotic pathogen; infected animals such as cats and dogs can transmit the virus to humans through bites. Rabies is endemic in over 150 countries, causes an estimated 59,000 deaths per year, and is almost 100% fatal after symptom onset [7,8]. Although no curative treatment exists, both pre-exposure prophylaxis and timely post-exposure vaccination are highly effective. Consequently, rabies vaccines are central to disease prevention [7,9].

Current vaccines are mainly inactivated vaccines (INVs) produced in chicken embryos, hamster kidney cells, human diploid cells and Vero cells [10]. Their main drawbacks are low productivity and modest immunogenicity, necessitating three doses for pre-exposure and four or five doses for post-exposure regimens [7,11]. To overcome these limitations, several groups have developed next-generation candidates. The recombinant G protein of RABV (RABV-G) or G + M virus-like particles (VLPs) have been successfully produced in yeast, HEK-293, BHK-21, CHO, insect and even plant cells [12,13,14]; however, additional research and technological advances are required before these vaccines can be licensed, and some fail to provide complete protection [13]. More efficacious rabies vaccines are therefore urgently needed.

The maturation of lipid nanoparticle (LNP) delivery systems has greatly facilitated the development of mRNA vaccines targeting the RABV-G, yielding robust humoral and cellular immune responses [15,16,17,18]. The key advantage lies in the mechanism of antigen presentation. mRNA vaccines, delivered via LNPs, are internalized by host cells where the encoded antigen is translated in vivo. This endogenously synthesized RABV-G undergoes natural post-translational modifications and is presented to the immune system through both the MHC-I and MHC-II pathways. This process mimics a natural viral infection, effectively stimulating not only potent humoral immunity (neutralizing antibodies, nAbs) but also critical CD4^+^ helper T cell (Th) and CD8^+^ cytotoxic T cell (CTL) responses, which are essential for clearing intracellular pathogens [19,20]. In contrast, exogenously delivered INVs and recombinant protein vaccines are primarily presented via the MHC-II pathway, leading to a dominant antibody response but a considerably weaker CD8^+^ CTL response.

The rabies virus N protein (RABV-N) sequence is highly conserved across strains, representing an excellent target for cross-protective immunity. During infection, it is abundantly expressed and efficiently processed through both MHC-I and MHC-II pathways, activating CD4^+^ and CD8^+^ T cells while promoting IFN-γ release. The inclusion of RABV-N thus broadens antigenic coverage, enhances the durability of cellular immunity, and provides an additional barrier against escape mutations in the surface RABV-G [21,22].

Although antibodies induced by RABV-N lack neutralizing activity, they function through the following mechanisms. First, enhancement of antibody-dependent cellular cytotoxicity (ADCC): during viral replication and assembly, viral proteins, including the RABV-N, are presented on the surface of infected cells. N-specific antibodies recognize these surface proteins. The Fc region of these antibodies then recruits immune cells, such as natural killer (NK) cells, thereby mediating the killing of virus-infected cells. This process eliminates the “virus factories” before widespread dissemination can occur. Second, promotion of antigen presentation and T cell activation: the binding of N antibodies to viral particles or infected cells forms antigen-antibody complexes. These complexes are more efficiently phagocytosed and processed by antigen-presenting cells, leading to a more robust activation of CD4^+^ and CD8^+^ T cells.

In this study we designed a composite-structured vaccine in which mRNA encoding the RABV-G (G-mRNA) and recombinant RABV-N expressed in *Escherichia coli* (*E. coli*) are co-encapsulated within dual-cationic LNPs to form a viral nucleocapsid-like nanostructure (NLS) vaccine. Assembly of this architecture was achieved with our proprietary dual-cationic LNP platform composed of two cationic lipids—DOTAP-Cl and DHA-1—together with the helper lipids mPEG-DTA-2K-1 and DOPC [23,24]. This vaccine utilizes LNPs to co-deliver the bivalent antigen formulation of RABV G-mRNA and RABV-N into cells. The G-mRNA is translated into RABV-G within the cytoplasm.

## 2. Materials and Methods

### 2.1. Cells

Human embryonic kidney 293T cells (Cat. No. CRL-3216) were purchased from ATCC, and hamster kidney BSR cells were obtained from the National Biomedical Cell Resource Bank of China. Cells were cultured in Dulbecco’s modified Eagle’s medium (DMEM; Thermo Fisher Scientific, Grand Island, NE, USA) supplemented with 10% (*v*/*v*) fetal bovine serum (FBS; Thermo Fisher Scientific, Grand Island, NE, USA) and 1% (*v*/*v*) penicillin–streptomycin (100 U mL^−1^ penicillin and 100 μg mL^−1^ streptomycin; Thermo Fisher Scientific, Grand Island, NE, USA).

### 2.2. Viruses

RABV challenge virus standard (CVS) strains CVS-11 [25] and CVS-24 [26] were kindly provided by Professor Zhongpeng Zhao of Shandong University. The CVS-24 strain was propagated in the brains of 3-week-old (10–12 g) SPF BALB/c mice, whereas the CVS-11 strain was propagated in BSR cells.

### 2.3. Animals

Except for CVS-24 virus propagation, which was performed in 3-week-old BALB/c mice, all other experiments were conducted using 4- to 5-week-old female specific-pathogen-free (SPF) BALB/c and Kunming (KM) mice. Immunizations were administered by hind-limb intramuscular injection, and euthanasia was performed by CO_2_ asphyxiation. All mice were purchased from Vital River Laboratory Animal Technology Co., Ltd. (Beijing, China).

The two mouse strains, KM and BALB/c, were selected for their complementary characteristics. The outbred KM mice model the genetic diversity of a human population, while the inbred BALB/c mice provide a uniform genetic background for highly reproducible results. The use of both strains enables a comprehensive evaluation of the vaccine’s immunogenicity.

### 2.4. Construction of the Recombinant Template Plasmid

To generate the G-mRNA template plasmid, the following elements (Table A1) were inserted between the HindIII (New England Biolabs, NEB, Ipswich, MA, USA) and BamHI (NEB, Ipswich, MA, USA) restriction sites of the pVAX1 vector: the 5′-untranslated region (UTR) from flavivirus, the Igκ secretory signal, a Kozak sequence, the RABV-G open reading frame (ORF), the 3′-UTR from flavivirus, and a poly(A) tail.

### 2.5. In Vitro Transcription (IVT)

The plasmid was linearized with BamHI and used as a template for IVT with T7 RNA polymerase. The 20 μL reaction mixture contained the following components: 2 μL T7 CleanCap Reagent AG Reaction Buffer (10×; NEB, Ipswich, NE, USA), 2 μL ATP (60 mM; NEB, Ipswich, NE, USA), 2 μL GTP (50 mM; NEB, Ipswich, NE, USA), 1 μL N1-Me-pUTP (100 mM; Synthgene, Nanjing, Jiangsu, China), 2 μL CTP (50 mM; NEB, Ipswich, NE, USA), 2 μL CleanCap Reagent AG (40 mM; NEB, Ipswich, NE, USA), 1 μg of linearized DNA template, 2 μL T7 RNA Polymerase Mix (NEB, Ipswich, NE, USA), and nuclease-free water to a final volume of 20 μL. The mixture was incubated at 37 °C for 2 h, Then, 2 μL of DNase I (NEB, Ipswich, NE, USA) was added and incubated at 37 °C for an additional 15 min to remove the DNA template, yielding the G-mRNA.

### 2.6. Transfection of 293T Cells

293T cells were transfected with G-mRNA using the transfection reagent Lipomaster 2000 (Vazyme, Nanjing, China). For each well of a 6-well plate, 2.5 μg of G-mRNA was combined with 5 μL of Lipomaster 2000 according to the manufacturer’s instructions. Wells treated with the transfection reagent alone were included as blank controls.

### 2.7. Sodium Dodecyl Sulfate–Polyacrylamide Gel Electrophoresis (SDS-PAGE) and Coomassie Brilliant Blue (CBB) Staining

SDS-PAGE was performed using a Bis-Tris SurePAGE pre-cast gel (4–20%; GenScript, Nanjing, China). Samples were diluted in 4× LDS Sample Buffer (GenScript, Nanjing, China), heated at 95 °C for 5 min, and loaded alongside a protein ladder (10–200 kDa; BioSharp, Beijing, China and 19–117kDa; Beyotime, Shanghai, China). Electrophoresis was carried out in 1× Tris-MOPS-SDS running buffer (GenScript, Nanjing, China) at 180 V for 50 min. After electrophoresis, the gel was soaked in CBB R-250 (Beyotime, Shanghai, China) staining solution for 30 min and then destained overnight to visualize the protein bands.

### 2.8. Western Blotting (WB)

Proteins were separated on 4–20% Bis-Tris gels and electro-transferred to 0.22 µm PVDF membranes (Merck Millipore, Darmstadt, Hesse, Germany). After blocking with 5% non-fat dry milk in TBST for 1 h at room temperature, the membranes were incubated overnight at 4 °C with primary antibodies diluted in 5% BSA-TBST. Following three 15-min washes with TBST, the membranes were incubated with HRP-conjugated secondary antibodies (1:10,000) for 1 h at room temperature. Immunoreactive bands were detected using enhanced chemiluminescence (ECL) and imaged with a ChemiDoc system (Bio-Rad, Hercules, CA, USA).

### 2.9. Recombinant Expression of RABV-N

A nucleotide sequence encoding RABV-N with an N-terminal 6× His tag (see Table A2) was inserted into the pET30a vector to generate the recombinant plasmid pET30a-RABV-N. The plasmid was transformed into BL21(DE3) competent *E. coli* cells (Thermo Fisher Scientific, Grand Island, NE, USA). Positive transformants were selected and cultured. When the optical density at 600 nm (OD_600_) reached 0.8, isopropyl β-D-1-thiogalactopyranoside (IPTG; Sigma-Aldrich, St. Louis, MO, USA) was added to a final concentration of 0.1 mM to induce protein expression at 16 °C for 24 h. The cells were harvested, lysed, and the debris was removed by centrifugation. The recombinant RABV-N protein was purified from the clarified supernatant by Ni Sepharose (Cytiva, Marlborough, MA, USA) affinity chromatography.

### 2.10. LNP Formulation and Encapsulation

The LNPs were composed of four lipid components—DHA-1, DOTAP-Cl, DOPC, and mPEG-DTA-1-2K—at a molar ratio of 36–40%:6–10%:16–20%:0.8–1.2%, together with 1.0–1.4% Span 85 and 0.4–0.6% Tween-80 [24]. An aqueous phase is prepared by mixing G-mRNA with RABV-N at a mass ratio of 4:1. The pre-formulated lipid phase and aqueous phase were homogenized to generate LNPs at a flow-rate ratio (FRR) of 1:3. The resulting nanoparticles were diluted 30- to 35-fold in a buffer containing 10 mM sodium acetate and 0.001% trehalose (pH 6.4), concentrated through a 100 kDa membrane, and then re-diluted into a solution of 20 mM sodium acetate, 0.01% trehalose, and 3.5% sucrose. After sterile filtration, the novel RABV NLS vaccine was obtained. The G-mRNA vaccine control was prepared without recombinant RABV-N.

### 2.11. RiboGreen Fluorescence Assay

The encapsulation efficiency of the G-mRNA and the NLS vaccine was determined using the Quant-iT RiboGreen RNA assay kit (Thermo Fisher Scientific, Grand Island, NE, USA) according to the manufacturer’s instructions.

### 2.12. Quality Control of LNP Vaccine

Dynamic light scattering (DLS) was employed to determine the particle size, polydispersity index (PDI) and zeta potential of the mRNA-LNP formulations. Samples were diluted 1:50 in 0.1× PBS (pH 7.4) to a final RNA concentration of approximately 0.1 mg mL^−1^ and measured at 25 °C using a Zetasizer Nano ZS instrument (Malvern Panalytical, Malvern, Worcestershire, UK). For size and PDI measurements, the instrument was operated in back-scatter mode (173°). zeta potential was determined by laser Doppler electrophoresis at 80 V using the Smoluchowski model.

### 2.13. Enzyme-Linked Immunosorbent Assay (ELISA)

RABV-G and RABV-N specific IgG levels were quantified using commercial ELISA kits (ACRO Biosystems, Beijing, China) according to the manufacturer’s protocol. Serum samples were subjected to two-fold serial dilution in the kit diluent, added to the wells in duplicate, and incubated for 1 h at 37 °C. After three washes with the supplied PBST, 100 µL of HRP-conjugated anti-species IgG detection antibody was added and incubated for 45 min at 37 °C. The wells were washed again, developed with 100 µL of TMB substrate for 10 min, and the reaction was stopped by adding 50 µL of 1 M H_2_SO_4_. Absorbance was measured at 450 nm with a reference wavelength of 630 nm.

### 2.14. Rapid Fluorescent Focus Inhibition Test (RFFIT)

A modified rapid fluorescent focus inhibition test (RFFIT) adapted to a 96-well microtiter plate platform was performed according to the WHO standard protocol [26]. Briefly, 50 µL of two-fold serially diluted serum (starting at a 1:10 dilution) was mixed with 50 µL of challenge virus (CVS-11, approximately 100 TCID_50_) and incubated for 90 min at 37 °C in 96-well plates. Next, 1.5 × 10^4^ BSR cells in 100 µL of complete DMEM were added per well, and the plates were incubated for 20 h at 37 °C under 5% CO_2_. After fixation with 80% acetone, the foci were stained with a fluorescein-conjugated anti-rabies nucleoprotein antibody (1:200 dilution; Abcam, Cambridge, Cambridgeshire, UK) for 1 h at 37 °C and counted under a fluorescence microscope. Each plate included virus control, cell control, and reference serum control wells. The neutralization titer was defined as the reciprocal of the highest serum dilution that resulted in a ≥50% reduction in fluorescent foci compared to the virus control.

### 2.15. Enzyme-Linked Immunospot Assay (ELISPOT)

An ELISPOT was performed using pre-coated mouse IFN-γ plates (Mabtech, Nacka, Stockholm County, Sweden). Splenocytes (2 × 10^5^ per well) were seeded in duplicate and stimulated with 5 μg mL^−1^ RABV-G and RABV-N peptide pools, concanavalin A (ConA, 2 μg mL^−1^; positive control), or medium alone (negative control) in 100 μL of complete RPMI-1640 (Thermo Fisher Scientific, Grand Island, NE, USA). After a 20 h incubation (37 °C, 5% CO_2_), the cells were removed, and the plates were developed sequentially with biotinylated anti-IFN-γ, streptavidin-ALP, and BCIP/NBT substrate according to the manufacturer’s instructions. Spots were counted using an ImmunoSpot reader with identical sensitivity settings. Data are presented as spot-forming units (SFU) per 10^6^ cells after subtracting the background (typically < 5 SFU). A positive T-cell response was defined as ≥2-fold above the background and ≥50 SFU per 10^6^ cells.

### 2.16. Quantitative Real-Time PCR (qPCR)

As previously described [17], RABV loads in tissues were determined by qPCR with absolute quantification, and cytokine expression by relative qPCR, using GAPDH as the internal reference (primers: GAPDH-F, AGGTCGGTGTGAACGGATTTG; GAPDH-R, TGTAGACCATGTAGTTGAGGTCA).

### 2.17. Data Statistics and Analysis

All statistical analyses were performed using SPSS 30.0 (IBM, Armonk, NY, USA) and GraphPad Prism 10.1 (GraphPad Software, San Diego, CA, USA) software. In significance testing, “ns” denotes no significant difference, * indicates *p* ≤ 0.05, ** indicates *p* ≤ 0.01, *** indicates *p* ≤ 0.001, and **** indicates *p* ≤ 0.0001.

## 3. Results

### 3.1. Preparation of G-mRNA of RABV

**Figure 1:**
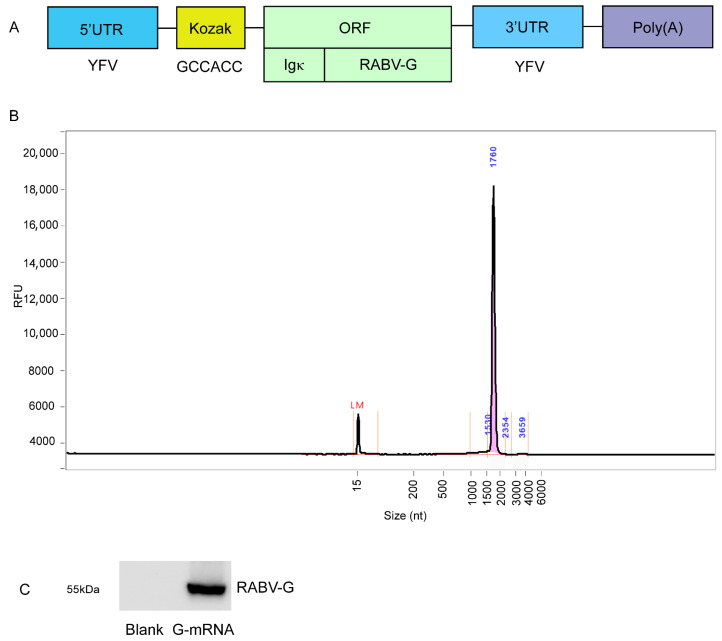
Construction and preparation of G-mRNA and expression of RABV-G in 293T cells. (**A**) The composition of recombinant template plasmid pVAX-RABV-G. (**B**) The purity of G-mRNA >95%. (**C**) G-mRNA can drive high-level expression of RABV-G in 293T cells, but no RABV-G expression was detected in blank control.

The recombinant template plasmid pVAX-RABV-G, which enables in vitro transcription of G-mRNA, was successfully constructed. The inserted elements—5′ UTR, Kozak sequence, Igκ secretory signal, RABV-G ORF, 3′ UTR and poly(A) tail—are shown in Figure 1A. The plasmid was linearized with BamHI and used as the template for G-mRNA synthesis. IVT with T7 RNA polymerase yielded G-mRNA with a purity of >95% (Figure 1B). The G-mRNA was transfected into 293T cells. After 24 h, cell lysates were prepared, and RABV-G was assessed by WB. Robust expression of RABV-G was clearly detected (Figure 1C).

### 3.2. Expression and Purification of RABV-N in E. coli

**Figure 2:**
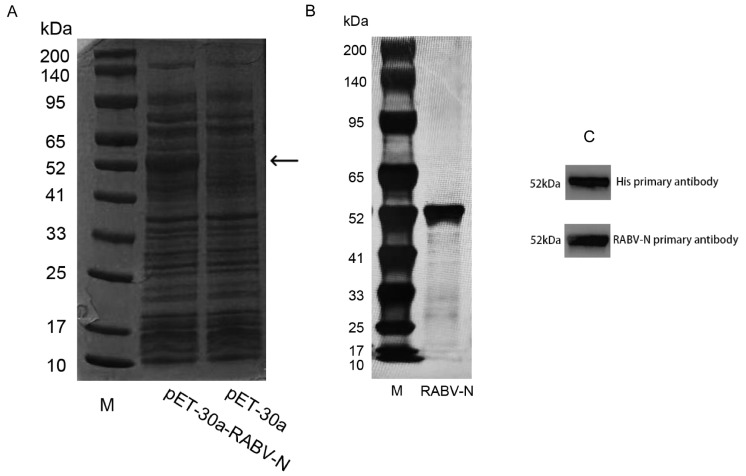
RABV-N was expressed in *E. coli*. (**A**) SDS-PAGE and CBB staining. *E. coli* containing the pET-30a-RABV-N plasmid showed a distinct recombinant protein band at 52 kDa, whereas the blank (cells transformed with empty pET-30a) exhibited no corresponding band (M: Marker). (**B**) SDS-PAGE and CBB staining. The 52 kDa RABV-N recombinant protein was obtained by His-affinity chromatography (M: Marker). (**C**) In WB analysis, specific bands at 52 kDa were detected using both anti-His and anti-RABV-N primary antibodies.

The RABV-N was expressed in *E. coli*. The cells were harvested by centrifugation at 1000× *g* and 4 °C for 20 min, washed three times with PBS (pH 7.4), resuspended, and disrupted by sonication. The supernatant of the bacterial lysate was collected after centrifugation at 8000× *g* and 4 °C for 30 min and then analyzed by SDS-PAGE followed by CBB staining. Compared with the blank control, a distinct band corresponding to RABV-N was observed at approximately 52 kDa (Figure 2A). The supernatant was subjected to His-tag affinity chromatography, yielding recombinant RABV-N with a purity of >90% (Figure 2B). The purified RABV-N was analyzed by WB using both anti-His and anti-N primary antibodies, each of which detected a single specific band (Figure 2C).

### 3.3. Quality Control of the LNP-Encapsulated RABV G-mRNA and NLS Vaccines

**Figure 3:**
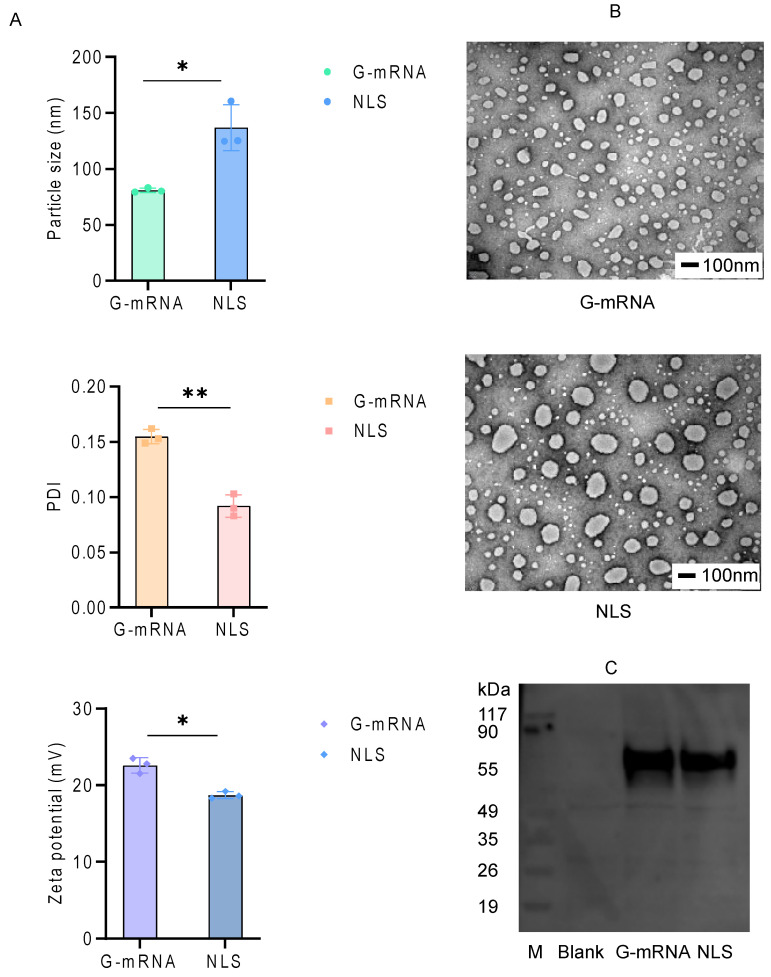
Quality control results of the encapsulated G-mRNA and NLS vaccine. (**A**) The particle size, PDI and zeta potential of G-mRNA and NLS vaccine (*n* = 3). * indicates *p* ≤ 0.05, ** indicates *p* ≤ 0.01. (**B**) Morphological examination of the G-mRNA and NLS vaccine by electron microscopy. (**C**) WB results of 293T cells transfected with empty LNPs (Blank), G-mRNA and NLS vaccine. G-mRNA, NLS vaccine can drive high-level expression of RABV-G in 293T cells, but no RABV-G expression was detected in blank control.

The LNP-encapsulated RABV G-mRNA vaccine and NLS vaccine were characterized for morphology, encapsulation efficiency, particle size, PDI, and zeta potential using negative-stain transmission electron microscopy (TEM), the RiboGreen fluorescence assay, and DLS. The results showed that both vaccines achieved an encapsulation efficiency of over 95%. In addition, the NLS vaccine exhibited a significantly larger particle size (136.9 nm vs. 80.9 nm), a lower PDI (0.09 vs. 0.15), and a lower zeta potential (+18.7 mV vs. +22.6 mV) compared to the G-mRNA vaccine (Figure 3A). Electron microscopy revealed that both the G-mRNA and NLS vaccines formed uniformly sized LNPs; however, the NLS vaccine particles were noticeably larger (Figure 3B). Both vaccines successfully transfected 293T cells, and Western blot analysis of cell lysates collected 24 h post-transfection detected a single, strongly expressed band for each (Figure 3C).

### 3.4. Determination of the Immunization Dose for the RABV G-mRNA Vaccine

**Figure 4:**
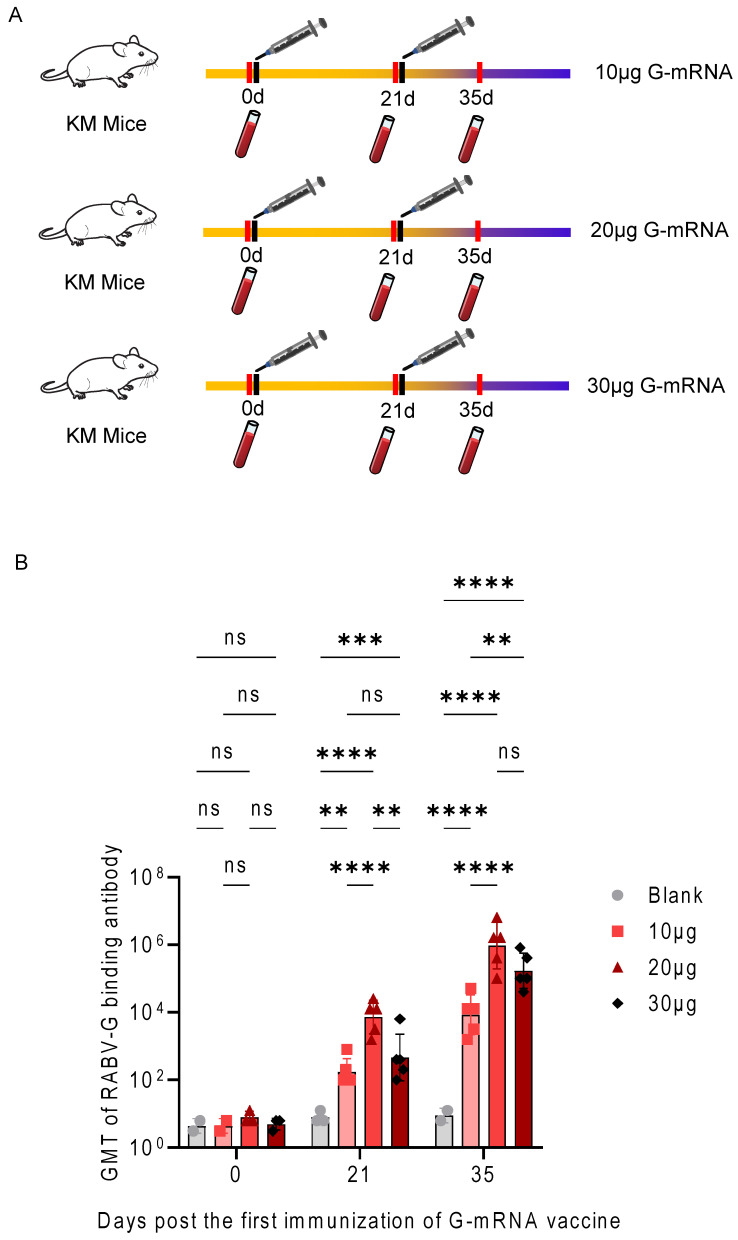
GMT of RABV-G induced by different doses of G-mRNA in immunized mice. (**A**) Schematic of the immunization protocol. KM mice (*n* = 5 per group) were immunized intramuscularly with 10 μg, 20 μg, or 30 μg of G-mRNA vaccine on days 0 and 21 (prime-boost regimen). Sera were collected on days 0, 21, and 35 for antibody analysis. (**B**) Kinetics of RABV-G-specific binding antibody responses. GMT of serum antibodies were determined by ELISA. All vaccinated groups showed a significant boost in antibody levels after the second immunization (day 35) compared to pre-immune levels (day 0) and the blank control group. The 20 μg dose group induced the highest antibody titers, followed by the 30 μg and 10 μg groups. ns indicates no significant difference, ** indicates *p* ≤ 0.01, *** indicates *p* ≤ 0.001, and **** indicates *p* ≤ 0.0001.

To determine the optimal dose of the G-mRNA vaccine, four groups of KM mice (*n* = 5 per group) were immunized on days 0 and 21 with empty LNPs, 10 μg, 20 μg, or 30 μg of G-mRNA. Serum samples were collected on days 0, 21 and 35 after the first immunization, and the titers of RABV-G-specific binding antibodies were determined by ELISA (Figure 4A). GMT were calculated from the ELISA data after log-transformation of individual values. An inter-group comparison of the three doses (10 μg, 20 μg and 30 μg) showed that on day 21 after the first immunization, the serum binding antibody titer in the 20 μg group was significantly higher than that in both the 10 μg and 30 μg groups, whereas no significant difference was observed between the 10 μg and 30 μg groups. By day 35, the 20 μg and 30 μg groups exhibited significantly higher titers than the 10 μg group, while the difference between the 20 μg and 30 μg groups remained non-significant (Figure 4B).

### 3.5. Dynamics of the Humoral Immune Response to RABV-G and RABV-N Induced by Different Immunization Schedules of the RABV NLS Vaccine

**Figure 5:**
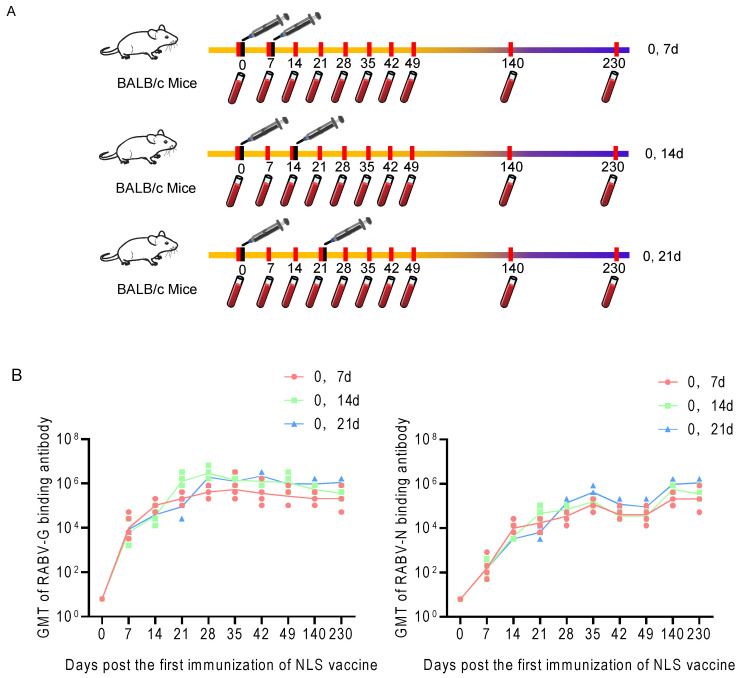
Kinetics of RABV-G and RABV-N binding antibody titers under different immunization schedules of RABV NLS vaccine. (**A**) RABV NLS vaccine was administered according to three different immunization strategies: days 0 and 7, days 0 and 14, and days 0 and 21. (**B**) Dynamic variation in humoral immune response of RABV-G and RABV-N GMT under the three different immunization strategies of NLS vaccine (*n* = 5).

Three groups of BALB/c mice (*n* = 5 per group) were immunized twice according to different schedules: days 0 and 7, days 0 and 14, or days 0 and 21. Serum samples were collected on days 0, 7, 14, 21, 28, 35, 42, 49, 140, and 230. RABV-G and RABV-N-specific binding antibody titers were measured by ELISA (Figure 5A). The results show that the 0, 7-day schedule elicited RABV-G and RABV-N antibody titers more rapidly, whereas the 0, 21-day schedule induces the highest peak titers; the 0, 14-day schedule produced intermediate results. Consequently, for pre-exposure prophylaxis, the 0, 21-day regimen is preferable, while for post-exposure prophylaxis the 0, 7-day regimen should be chosen to accelerate the immune response (Figure 5B).

### 3.6. Neutralizing Antibody Response to the RABV NLS Vaccine Measured by RFFIT

**Figure 6:**
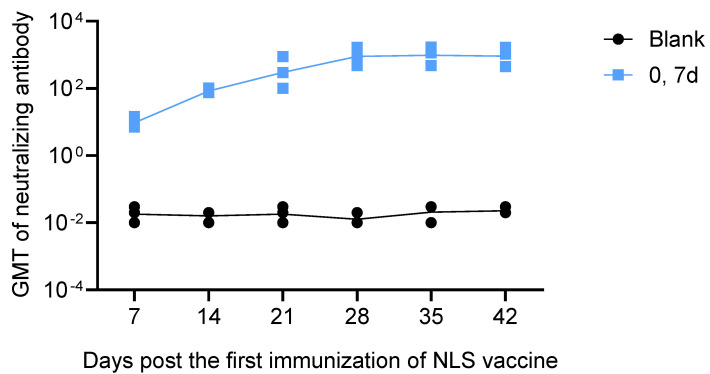
Kinetics of neutralizing antibody titers in mice immunized on days 0 and 7 (*n* = 3). A single dose of the NLS vaccine induced neutralizing antibody titers of approximately 10 IU mL^−1^ within 7 days. 7 days after the second dose (day 14 post-prime), neutralizing antibody titers rose to 85 IU mL^−1^, and the peak titer after the two-dose course reached 1000 IU mL^−1^. The neutralizing antibody titer in mice immunized with empty LNPs (Blank group) was below 0.5 IU mL^−1^ on days 7, 14, 21, 28, 35 and 42.

Although ELISA detected high titers of binding antibodies induced by the vaccine, these antibodies reflect only the binding capacity to the viral antigen and do not directly demonstrate antiviral activity. Thus, we further conducted a neutralizing antibody assay. Two groups of BALB/c mice (*n* = 3 per group) were immunized with the NLS vaccine or empty LNPs on days 0 and 7. The kinetics of neutralizing antibody titers were measured by RFFIT on days 7, 14, 21, 28, 35, and 42. The results showed that a single dose of the NLS vaccine induced neutralizing antibody titers of approximately 10 IU mL^−1^ within 7 days, a value 20 times greater than the threshold of 0.5 IU mL^−1^, which is an accepted indicator of an adequate vaccination response [10]. 7 days after the second dose (day 14 post- primary immunization), neutralizing antibody titers rose to 85 IU mL^−1^, and the peak titer after the two-dose course reached 1000 IU mL^−1^ (Figure 6).

### 3.7. Specific CTL Response Profiling Elicited by the RABV NLS Vaccine, G-mRNA Vaccine and INV

**Figure 7:**
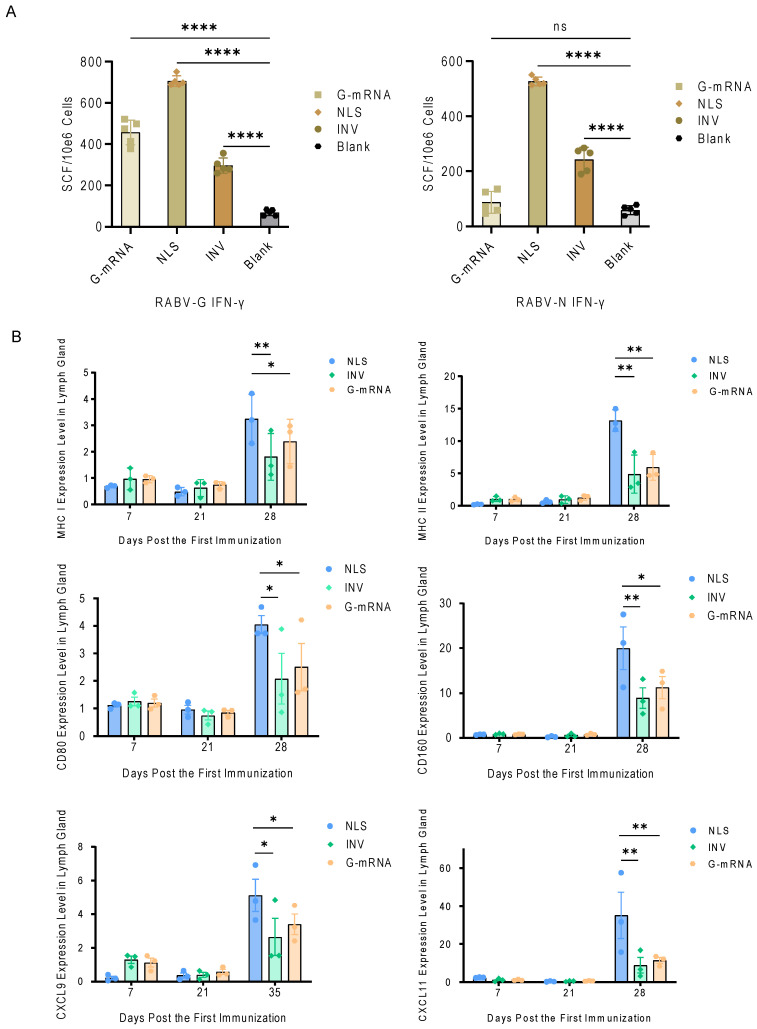
Activation of antigen-specific immune responses after NLS or INV vaccination in mice. (**A**) G antigen-specific T cell responses evaluated by ELISpot. Following two doses of NLS and G-mRNA vaccine immunization on days 0 and 21, and three doses of INV immunization on days 0, 7 and 21, splenocytes from BALB/c mice (*n* = 5 per group) were assessed for IFN-γ secretion. The NLS vaccine induces the most potent response compared to G-mRNA and INV. ns indicates no significant difference, and **** indicates *p* ≤ 0.0001. (**B**) Activation of cellular immunity in draining lymph nodes. BALB/c mice (*n* = 3 per group) were immunized with NLS, G-mRNA, or INV on different schedules. Draining lymph nodes were harvested on days 7, 21, and 28 post-primary immunization. The mRNA expression levels of MHC-I, MHC-II, CXCL9, CXCL11, CD80, and CD160 were quantified by qPCR. On day 28, all six genes were significantly up-regulated in the NLS vaccine group compared to the other groups. * indicates *p* ≤ 0.05, and ** indicates *p* ≤ 0.01.

To evaluate the strength of vaccine-induced antigen-specific CTL responses, 4 groups of BALB/c mice (*n* = 5 each) were used. Animals in the G-mRNA and NLS vaccine groups received two immunizations on days 0 and 21, whereas the INV group was immunized three times on days 0, 7, and 21; the blank control group received empty LNPs only on days 0 and 21 (see Table 1). All mice were euthanized on day 7 after the last dose. Spleens were aseptically removed, and splenocytes were isolated. Antigen-specific IFN-γ-secreting T cells were enumerated by ELISpot. The results showed that for the G antigen, NLS vaccine elicited the strongest specific response, followed by the G-mRNA vaccine, while INV induced a comparatively weaker reaction. Regarding the N antigen, the G-mRNA group—lacking the RABV-N component—showed the lowest response; the NLS vaccine again produced the highest reactivity, and the INV response fell between the two (Figure 7A).

To compare the activation of cellular immunity elicited by the different vaccines, three groups of BALB/c mice (*n* = 3 per group) received the NLS vaccine, INV, or G-mRNA vaccine according to their respective immunization schedules. Mice in the G-mRNA and NLS vaccine groups received two immunizations on days 0 and 21, whereas the INV group was immunized three times on days 0, 7, and 21. Lymph nodes were harvested on days 7, 21, and 28 after the first immunization, and expression of MHC-I, MHC-II, CXCL9, CXCL11, CD80, and CD160 was quantified by qPCR. Results showed that on day 28 (i.e., 7 days after the boost), all six genes were expressed at significantly higher levels in the NLS vaccine group than in the INV and G-mRNA vaccine groups (Figure 7B).

### 3.8. Challenge Protection Assay with RABV CVS-24 Strain

Four groups of KM mice (*n* = 22 per group; 10 mice per group were used for the challenge protection test, and 12 were used for the viral load kinetics assay) were immunized according to the following schedules: the NLS vaccine and G-mRNA vaccine groups received two doses on days 0 and 21; the INV group received three doses on days 0, 7, and 21; and the blank control group was administered empty LNPs on days 0 and 21 (see Table 2).

**Figure 8:**
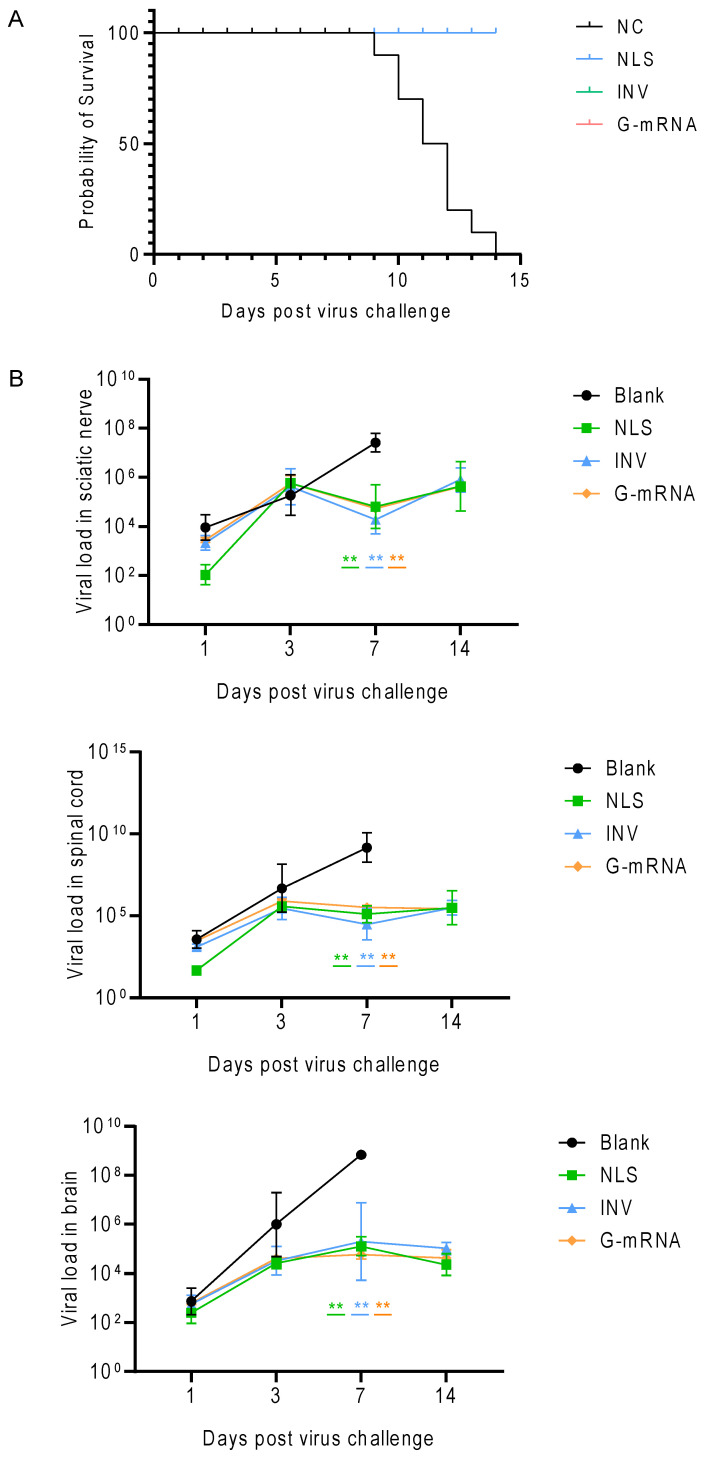
Survival rate and viral-load kinetics in different tissues of mice challenged with the CVS-24 strain of RABV. (**A**) After two doses of the NLS and G-mRNA vaccine on days 0 and 21, and three doses of the INV on days 0, 7, and 21, challenge with 25 LD_50_ CVS-24 showed that all of the three vaccines provided 100% protection (*n* = 10). (**B**) Viral load in tissues after virus challenge. Mice (*n* = 3 per group per time point) were euthanized on days 1, 3, 7, and 14 post-challenge. Viral loads in sciatic nerve, spinal cord, and brain were quantified by qPCR. On day 7, the blank control group had markedly higher viral loads than all vaccine groups. By day 14, all blank control mice had succumbed, while all vaccinated mice survived with low viral loads. ** indicate *p* ≤ 0.01. The green line indicates the NLS group compared with the blank group; the orange line, the G-mRNA group; and the blue line, the INV group on day 7 (all versus the blank group).

On day 35 after the first immunization, all mice were challenged intramuscularly with 25 LD_50_ of the RABV CVS-24 strain. Survival was monitored daily. The results showed that the NLS vaccine, INV, and G-mRNA vaccine groups conferred 100% protection to the mice, whereas all animals in the blank control group succumbed between days 9 and 14 post-challenge (Figure 8A).

After the virus challenge, three mice per group were euthanized on days 1, 3, 7, and 14. The sciatic nerve, spinal cord, and brain were collected. Total RNA was extracted, and the viral load was quantified by qPCR. The results showed that the viral loads in the three tissues did not differ significantly on days 1 and 3 post challenge. On day 7, however, the blank control group had markedly higher viral loads in all three tissues than the NLS vaccine, INV and G-mRNA vaccine groups. By day 14, all animals in the blank control group had died, whereas all mice in the NLS vaccine, INV and G-mRNA vaccine groups survived and displayed no appreciable change in the viral load in the three tissues (Figure 8B).

## 4. Discussion

**Figure 9:**
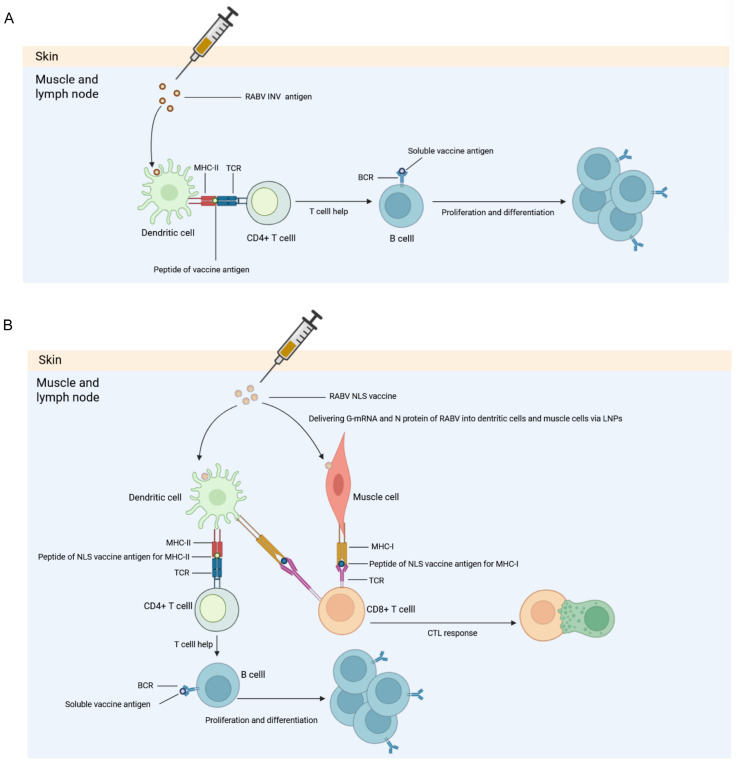
Mechanisms of immune activation by RABV INV and NLS vaccine. (**A**) RABV INV: Upon administration, the vaccine antigens are taken up by dendritic cells. Antigens are processed and presented via the MHC-II pathway, leading to the activation of CD4^+^ T cells. These T cells provide help to B cells, promoting their proliferation and differentiation into antibody-producing plasma cells, which secrete nAbs against the viral antigen. (**B**) RABV NLS vaccine: The LNPs co-delivering G-mRNA and RABV-N target antigen-presenting cells and muscle cells. The G-mRNA is translated intracellularly, and the synthesized antigens are presented via the MHC-I pathway, robustly activating CD8^+^ CTLs. Concurrently, antigens are also presented via the MHC-II pathway to engage CD4^+^ T cells help for B cell activation. This dual-pathway activation induces a comprehensive immune response comprising both potent nAbs and strong cellular immunity.

In contrast to the INV, which primarily activates the MHC-II pathway (Figure 9A), our NLS vaccine enables the simultaneous activation of both MHC-I and MHC-II immune pathways (Figure 9B). Our findings demonstrate that the NLS vaccine induces a more robust and balanced immune activation than the INV. This dual activation of MHC-I and MHC-II pathways is crucial for generating synergistic nAbs and CD8^+^ CTL activity, which may translate to superior protection.

Our previous studies suggested that dual-cationic lipid formulation can not only encapsulate mRNA alone but also simultaneously entrap both mRNA and protein or allow protein to be anchored on the LNP surface after mRNA loading [23,24]. Based on this proprietary dual-cationic lipid technology, we designed the rabies virus NLS vaccine, which is no longer a simple G-mRNA vaccine, but a novel modal that co-encapsulates G-mRNA together with the RABV-N.

This design is based on two rationales. First, the RABV-G is the key antigen responsible for eliciting nAbs; in addition to inducing specific nAbs, it effectively stimulates robust CD4^+^ and CD8^+^ T cell immunity. Second, RABV-N is one of the most conserved structural proteins across members of the *Lyssavirus* genus, with >90% amino acid identity among classical RABV strains [27,28,29]. Therefore, the B cell and T cell immunity induced by RABV-N is conserved across different strains. Antigen-presenting cells such as dendritic cells (DCs) and macrophages, internalize RABV-N and present RABV-N-derived peptides on MHC-II, triggering CD4^+^ T cell responses [30,31].

CD4^+^ T cells are the master helpers of vaccine-induced immunity. Upon activation, they differentiate into specialized subsets that drive the clonal expansion of CD8^+^ CTL, B cell affinity maturation, and long-lived antibody production. In the absence of this help, both primary CD8^+^ T cell responses and B cell memory are severely impaired, whereas the presence of CD4+ T cell help correlates with durable protection after vaccination [32].

The CD8^+^ CTL response is indispensable for clearing infected cells, controlling viral spread, and providing long-term protective immunity [33,34]. Upon recognition of viral peptides presented by MHC-I molecules, CD8^+^ CTLs exert antiviral activity through two parallel mechanisms: cytolytic activity, involving the release of perforin and granzyme to induce apoptosis of infected cells and prevent the production of progeny virus; and non-cytolytic suppression, mediated by the secretion of IFN-γ and TNF-α, which inhibit viral gene expression and prime neighboring cells to adopt an antiviral state. Conversely, viruses that down-regulate MHC-I expression or generate CD8^+^ CTL escape mutations achieve persistence, underscoring that a robust CD8^+^ CTL response is as essential as nAbs for durable protection. Therefore, next-generation vaccines should designed to deliberately engage both arms of adaptive immunity to maximize efficacy.

In this study, we measured the expression levels of MHC-I and CD160—molecules linked to CD8^+^ CTL responses—after the second dose of the NLS vaccine. Both were significantly higher than those in the INV group, indicating that the NLS vaccine elicits a more robust CD8^+^ CTL response. The expression of MHC-II (associated with CD4^+^ T cells) and CD80 (involved in the activation of both CD4^+^ and CD8^+^ T cells) was also elevated relative to the INV group, demonstrating that the NLS vaccine induces a stronger CD4^+^ T cell response as well.

In this study, we also measured the vaccine-induced expression of CXCL9 and CXCL11. Both chemokines remained significantly higher 7 days after the second dose of the NLS vaccine compared to the INV group, implying enhanced generation or reactivation of memory-precursor cells, thereby establishing a foundation for longer-lasting protection.

CXCL9 and CXCL11 are IFN-γ–inducible chemokines that act through CXCR3 receptor to orchestrate adaptive immunity. chemokines establish a chemotactic gradient that draws CXCR3^+^ naïve and activated CD4^+^ and CD8^+^ T cells from the bloodstream into draining lymph nodes and peripheral tissues, ensuring that effector cells reach the sites of antigen presentation. In addition, persistent CXCL9 and CXCL11 signaling during T-cell expansion phase promotes the differentiation of central memory CD4^+^ and CD8^+^ T cells [35,36,37].

Compared with the G-mRNA vaccine and INV, the NLS vaccine elicits significantly stronger humoral and cellular immune responses. Seven days after a single dose, the NLS vaccine elicited neutralizing antibody titers of approximately 10 IU mL^−1^ Following the secondary immunization, the neutralizing antibody titer reached 1000 IU mL^−1^, accompanied by a robust specific CD8^+^ CTL response, demonstrating superior potency to both the G-mRNA vaccine and INV. After two doses administered on days 0 and 21, 100% of the mice survived a challenge with 25 LD_50_ of the fixed RABV strain at day 14 post-boost.

mRNA-based vaccines generally exhibit a favorable safety profile, characterized by their non-integrating nature and transient antigen expression, which eliminates the risk of genomic integration. Common adverse events, such as injection-site reactions and transient systemic symptoms (e.g., fever and fatigue), are typically mild to moderate and self-limiting. These reactogenicities are largely attributed to innate immune activation induced by both the LNPs and the intrinsic immunostimulatory properties of mRNA [38,39,40].

Recombinant protein vaccines are renowned for their favorable safety profile. As they contain only purified, specific antigenic fragments without genetic material, they cannot cause infection, thereby avoiding risks associated with live-attenuated vaccines. Their well-defined composition minimizes reactogenicity, resulting primarily in mild local or systemic adverse events. The established safety record of licensed vaccines, such as those for hepatitis B and human papillomavirus (HPV), provides a strong foundation for newer candidates [41,42].

For novel mRNA-protein combination vaccines, such as one co-delivering RABV G-mRNA and RABV-N protein, additional considerations arise. The simultaneous introduction of multiple antigenic modalities may lead to enhanced reactogenicity or unforeseen synergistic effects, necessitating rigorous evaluation of local and systemic responses. Furthermore, the potential for altered immune kinetics or antigenic immunodominance requires careful investigation to ensure a balanced and effective immune response. Preclinical and clinical studies must thoroughly assess these aspects to confirm the safety of such advanced vaccine platforms.

Certainly, the NLS vaccine also has its drawbacks. First, while mRNA vaccines are characterized by a short development cycle, the addition of a protein to construct the NLS formulation prolongs this timeline. Second, the inclusion of the recombinant protein introduces extra quality control items and increases analytical complexity. Nevertheless, for rabies vaccines—where conventional products already exist—in our view, the additional time required to achieve superior immunogenicity is worthwhile.

## 5. Conclusions

In this study, we developed a novel RABV NLS vaccine using a dual-cationic LNP platform, co-encapsulating RABV G-mRNA and recombinant RABV-N to simultaneously engage MHC-I and MHC-II pathways. The NLS vaccine demonstrated significantly enhanced immunogenicity, inducing robust humoral and cellular immune responses—including high nAb titers (peaking at 1000 IU mL^−1^), potent CD8^+^ CTL and CD4^+^ Th activation—and provided 100% protection against a lethal RABV challenge with rapid antibody induction and no viral rebound. By activating both MHC-I and MHC-II pathways, the dual-antigen design mimicked natural infection and promoted synergistic immunity, while the inclusion of RABV-N broadened antigenic coverage and strengthened T cell responses against potential viral escape. The vaccine maintained a favorable safety profile akin to conventional mRNA vaccines, with transient and mild reactogenicity. These findings highlight the NLS platform as a promising next-generation rabies vaccine candidate with potential applicability to other viral diseases, warranting further optimization, long-term immunity evaluation, and clinical translation.

## Figures and Tables

**Table 1:** Immunogens and immunization schedules for the different BALB/c mice groups (*n* = 8).

Group Name	Group Size (*n*)	Immunogen	Immunization Schedule
G-mRNA	*n* = 8, 5 for ELISpot, 3 for qPCR	20 μg G-mRNA	0, 21
NLS	*n* = 8, 5 for ELISpot, 3 for qPCR	20 μg G-mRNA and 5 μg RABV-N	0, 21
INV	*n* = 8, 5 for ELISpot, 3 for qPCR	INV	0, 7, 21
Blank	*n* = 8, 5 for ELISpot, 3 for qPCR	Empty LNPs	0, 21

**Table 2:** Immunogens and immunization schedules for the different KM mice groups (*n* = 22).

Group Name	Group Size (*n*)	Immunogen	Immunization Schedule
G-mRNA	*n* = 22, 10 for the challenge protection test, 12 for the viral load kinetics assay	20 μg G-mRNA	0, 21
NLS	*n* = 22, 10 for the challenge protection test, 12 for the viral load kinetics assay	20 μg G-mRNA and 5 μg RABV-N	0, 21
INV	*n* = 22, 10 for the challenge protection test, 12 for the viral load kinetics assay	INV	0, 7, 21
Blank	*n* = 22, 10 for the challenge protection test, 12 for the viral load kinetics assay	Empty LNPs	0, 21

## Data Availability

All data supporting the findings of this study are available within the article. Additional datasets and raw data generated during this research are available from the corresponding author upon reasonable request.

## Abbreviations

The following abbreviations are used in this manuscript:

RABV	Rabies virus
G	glycoprotein
M	matrix protein
P	phosphoprotein
L	RNA polymerase
N	nucleoprotein
INV	inactivated vaccine
RABV-G	G protein of rabies virus
VLP	virus-like particle
LNP	lipid nanoparticle
nAb	neutralizing antibody
Th	helper T cell
CTL	cytotoxic T cell
G-mRNA	mRNA encoding the G protein
RABV-N	N protein of rabies virus
ADCC	antibody-dependent cellular cytotoxicity
NK	natural killer
NLS	nucleocapsid-like nanostructure
DMEM	Dulbecco’s modified Eagle medium
FBS	fetal bovine serum
CVS	challenge virus standard
SPF	specific-pathogen-free
KM	Kunming
SDS-PAGE	sodium dodecyl sulfate—polyacrylamide gel electrophoresis
CBB	Coomassie brilliant blue
WB	Western blotting
IPTG	isopropyl β-D-1-thiogalactopyranoside
UTR	untranslated region
ORF	open reading frame
IVT	in vitro transcription
ECL	enhanced chemiluminescence
FRR	flow-rate ratio
DLS	dynamic light scattering
PDI	polydispersity index
ELISA	enzyme-linked immunosorbent assay
GMT	geometric mean titers
RFFIT	rapid fluorescent focus inhibition test
ELISPOT	enzyme-linked immunospot assay
SFU	spot-forming units
qPCR	quantitative real-time PCR
TEM	transmission electron microscopy
DC	dendritic cell

## Appendix A

**Table A1:** Composition of the G-mRNA template.

Sequence Name	Sequence Length (bp/AA)	Sequence
5′-UTR from flavivirus	118	agtaaatcctgtgtgctaattgaggtgcattggtctgcaaatcgagttgctagg caataaacacatttggattaattttaatcgttcgttgagcgattagcagagaact gaccagaac
Kozak sequence	6	gccacc
Igκ secretory signal	21	METDTLLLWVLLLWVPGSTGD
RABV-G ORF	439	KFPIYTIPDKLGPWSPIDIHHLSCPNNLVVEDEGCTN LSGFSYMELKVGYISAIKVNGFTCTGVVTEAETYTNF VGYVTTTFKRKHFRPTPDACRSAYNWKMAGDPRYE ESLHNPYPDYHWLRTVKTTKESVVIISPSVADLDPYD KSLHSRVFPRGKCSGITVSSAYCSTNHDYTIWMPENP RLGTSCDIFTNSRGKRASKGSKTCGFVDERGLYKSLK GACKLKLCGVLGLRLMDGTWVAIQTSNETKWCPP DQLVNLHDFHSDEIEHLVVEELVKKREECLDALESI MTTKSVSFRRLSHLRKLVPGFGKAYTIFNKTLMEAD AHYKSVRTWNEIIPSKGCLRVGGRCHPHVNGVFFN GIILGPDGHVLIPEMQSSLLQQHMELLESSVIPLMHP LADPSTVFKDGDEVEDFVEVHLPDVHKQVSGVDLG LPNWGK
3′-UTR from flavivirus	123	gacactgttccaagcaacaaccaagaactagaccttgtacataggacaaaact gaaaccgggataaaaaccacggatggagaaccggactccacacatcaaaca gaagaggatgtcagcccag
Poly(A) tail	138	aaaaaaaaaaaaaaaaaaaaaaaaaaaaaaaaaaaaaaaagcatatgact aaaaaaaaaaaaaaaaaaaaaaaaaaaaaaaaaaaaaaccgcgtgctgaa aaaaaaaaaaaaaaaaaaaaaaaaaaaaaaaaaaaaaa

**Table A2:** RABV-N sequence.

Sequence Name	Sequence Length (AA)	Amino Acid Sequence
6× His Tag	6	HHHHHH
N-protein	449	DADKIVFKVNNQVVSLKPEIIVDQYEYKYPAI KDLKKPCITLGKAPDLNKAYKSVLSGMNAA KLDPDDVCSYLAAAMQFFEGTCPEDWTSYG ILIARKGDRITPNSLVEIKRTDVEGNWALTGG MELTRDPTVSEHASLVGLLLSLYRLSKISGQN TGNYKTNIADRIEQIFETAPFVKIVEHHTLMT THKMCANWSTIPNFRFLAGTYDMFFSRIEHL YSAIRVGTVVTAYEDCSGLVSFTGFIKQINLT AREAILYFFHKNFEEEIRRMFEPGQETAVPHS YFIHFRSLGLSGKSPYSSNAVGHVFNLIHFVG CYMGQVRSLNATVIAACAPHEMSVLGGYLG EEFFGKGTFERRFFRDEKELQEYEAAELTKSD VALADDGTVNSDDEDYFSGETRSPEAVYTRI MMNGGRLKRSHIRRYVSVSSNHQARPNSFA EFLNKTYSNDS
